# DEHyART trial: Study protocol for phase 2 randomised controlled study assessing the role of dose escalation using [18F] fluoromisonidazole positron emission tomography/computed tomography in head and neck cancers

**DOI:** 10.3332/ecancer.2025.1937

**Published:** 2025-07-02

**Authors:** Sarthak Tandon, Manoj Gupta, Parveen Ahlawat, Madhur Verma, Apoorva Nayak, Akash Bellige, Kundan S Chufal, Jaskaran S Sethi, Anjali Pahuja, Shreya Rai, Abhishek Singh, Vikas Arora, Vishal Yadav, David K Simson, Irfan Ahmad, Sandeep Singh, Dipesh Vashisht, Azhar Ansari, Rashmi Bansal, Abhishek Bhadri, Harsh Vyas, Manindra Mishra, Rajat Saha, Mudit Agarwal, Partha S Chowdhary, Ajay K Dewan, Munish Gairola

**Affiliations:** 1 Department of Radiation Oncology, Rajiv Gandhi Cancer Institute and Research Centre, Delhi 110085, India; 2 Department of Nuclear Medicine, Rajiv Gandhi Cancer Institute and Research Centre, Delhi 110085, India; 3 Department of Community and Family Medicine, AIIMS, Bhatinda 110029, India; 4 Department of Surgical Oncology, Rajiv Gandhi Cancer Institute and Research Centre, Delhi 110085, India; 5 Division of Medical Physics, Department of Radiation Oncology, Rajiv Gandhi Cancer Institute and Research Centre, Delhi 110085, India; 6 Department of Medical Oncology, Rajiv Gandhi Cancer Institute and Research Centre, Delhi 110085, India; a https://orcid.org/0000-0002-2319-0391

**Keywords:** dose escalation, IMRT, HNSCC, radiotherapy, hypoxia, FMISO

## Abstract

**Background:**

Head and neck squamous cell carcinoma is often treated with radiotherapy, frequently combined with chemotherapy, to improve overall survival (OS). Despite advancements, locoregional control (LRC) remains a significant challenge, with 15%–50% of patients experiencing locoregional recurrence, negatively impacting OS and quality of life. Hypoxia within tumor cells is a critical factor contributing to treatment failure, necessitating higher radiation doses to achieve similar therapeutic effects as in normoxic cells. This study aims to investigate the role of dose escalation using [18F] fluoromisonidazole (FMISO) positron emission tomography/computed tomography (PET CT) to target hypoxic sub-volumes in head and neck cancer (HNC) to improve LRC.

**Methods:**

The dose-escalated hypoxia-adjusted radiotherapy trial is an open-label, parallel, randomised, single-centre, phase II study. Patients with HNC will undergo [18F]. FMISO PET CT to identify hypoxic regions. Normoxic patients will be labeled as Arm 1 and will not be part of the primary assessment. Patients with hypoxia will be stratified into two arms (2 and 3). Arm 2 will receive standard radiotherapy of 70 Gy in 2 Gy fractions, while Arm 3 will receive an additional boost to the hypoxic sub-volumes, delivering a total of 80 Gy (Phase 2). All patients in Arms 2 and 3 will also receive concurrent chemotherapy with cisplatin. Patients will be monitored weekly for treatment tolerance, with acute adverse events recorded according to National Cancer Institute Common Terminology Criteria for Adverse Events v5.0. The primary endpoint is LRC, defined as the time from randomisation to the first histopathologically confirmed relapse of locoregional disease. Secondary endpoints include OS, locoregional relapse-free survival, acute and late toxicity and patient-reported outcomes assessed using the European Organisation for Research and Treatment of Cancer QLQ-C30 and QLQ-H&N35 questionnaires.

**Discussion:**

This study addresses a critical gap in the management of HNC by targeting hypoxic regions within tumours, potentially improving LRC and, consequently, OS. The use of [18F] FMISO PET CT for identifying hypoxic sub-volumes allows for tailored radiation dose escalation, which could overcome the radioresistance associated with hypoxia. By comparing outcomes among standard radiotherapy (Arm 2) and dose-escalated treatment (Arm 3), this trial aims to establish a more effective therapeutic strategy for HNC patients.

**Trial registration:**

This trial is registered with the Clinical Trials Registry of India (CTRI/2024/04/065373), registered on 08^th^ April 2024 on ctri.nic.in and clinicaltrials.gov (NCT06087614) registered on 18th September 2023 on clinicaltrials.gov.

## Introduction

### Background and rationale

Radiotherapy is the mainstay in treating head and neck squamous cell carcinoma (HNSCC). The concurrent chemotherapy with radiotherapy gives an additional absolute survival benefit of 6.5% at 5 years [1]. Despite advancements in radiation and chemotherapy, the overall survival (OS) in HNSCC has been dismal. Locoregional control (LRC) drives the overall survival in most head and neck squamous cell carcinoma, however, it is reported that about 15%–50% of patients fail locoregionally [2,3,4].

Locoregional recurrence not only causes a drop in OS but is also a major concern for adding overall morbidity and deteriorating patients’ quality of life. Therefore, further improving on LRC would be a prudent therapeutic strategy for increasing OS and enhancing the quality-of-life indices. The authors assessed the pattern of failure for HNSCC being treated with intensity-modulated radiotherapy (IMRT). They found that with a 3-year LRC of 48.9%, most failed in the high-dose region (69.2%), suggesting inherent biological resistance to treatment [5,6]. One hypothesised reason for this treatment failure is the presence of hypoxia in tumour cell lines, which may necessitate three times the radiation dose to achieve an isotherapeutic effect comparable to that in normoxic cells [7].

### Hypoxia in head and neck patients

Tumour hypoxia is a major risk factor for local and distant failure after radiotherapy [8]. It is reported that up to 80% of HNSCC have baseline hypoxia, with sites like hypopharyngeal and oropharyngeal having a higher incidence of hypoxia than the other sites of the head and neck [9]. Non-invasive hypoxia assessment in head and neck cancer (HNC) has been extensively conducted using various radioactive tracers such as [18F] fluoromisonidazole (FMISO), [18F] fluoroazomycin-arabinoside (FAZA) and [18F] HX4. These have been individually studied in various studies [10–14] and have been correlated with other hypoxia biomarkers in numerous studies as well [15–17]. A recent meta-analysis showed that positron emission tomography (PET)-measured hypoxia with FMISO or FAZA strongly impacted OS and LRC in HNSCC. Most trials have used FAZA and FMISO, which have shown equivalence in multicentre trials [18]. The meta-analysis by Zschaeck *et al* [18]. showed that the hypoxia PET parameter was an independent risk factor in multivariate analysis, irrespective of any established clinical parameter. Studies have investigated whether subsequent recurrences overlap with the initial hypoxic volumes, yielding discordant results. Boeke *et al* [19]. reported a high median overlap of 42%, while two other studies [20, 21] found no significant overlap. Nishikawa *et al* [22] demonstrated that an initial tissue-to-muscle ratio (TMR) greater than 2.42 could predict failure in terms of spatial location. These findings underscore the need for a second FMISO scan during the course of radiotherapy, which may better predict chronic hypoxia. Considering the significant influence of tumour-associated hypoxia on the response of HNSCCs to radiotherapy, different treatment strategies have been proposed and tested in exploratory analyses, trying to identify the stability of these sub-volumes over multiple fractions and the possibility of escalating treatment doses to high-risk hypoxic sub-volumes (HSVs) [23–25].

### Rationale for dose escalation in HNC

Theoretical planning studies have been conducted to assess the dose escalation to the biology-selected FMISO volumes [26–28]. Thorwarth *et al* [27] predicted the increase in tumour control probability from 55.9% to 70.9% by increasing the dose up to 84 Gy to the FMISO-selected hypoxic volumes. In a recent Phase II randomised study by Welz *et al* [29], escalated doses to the hypoxic volumes determined by FMISO. The authors escalated doses to this volume to 77 Gy (2.2 Gy per fraction) by the simultaneous integrated boost technique and compared it to standard fractionation and doses. There was a non-significant increase in 5-year local control favouring dose dose-escalated arm. However, the study was prematurely closed due to poor accrual. Another flaw in the study design could be a marginal increase in dose escalation, with a 2% mean dose escalation against a planned 10% dose escalation. The mean hypoxic volume subjected to dose escalation was 2.9 ml, which might be too small to make any significant difference in outcomes. An ongoing phase III trial, ESCALOX [30], assesses dose escalation to the hypoxic volumes to a dose of 80.5 Gy and is currently accruing patients.

### Objectives

There exists a critical need to increase LRC in HNC while maintaining the patients’ quality of life. Numerous studies [31–33] have investigated dose and volume modifications in p16-positive oropharyngeal cancers. However, there is a paucity of literature addressing these modifications in p16-negative oropharyngeal and non-oropharyngeal tumours, underscoring a significant research gap. The primary objective of this study – DEHyART is to assess the role of dose escalation using [18F]. FMISO positron emission tomography/computed tomography (PET CT) in HNC to improve LRC by targeting HSV. Specifically, the study aims to evaluate whether increasing the radiation dose to hypoxic areas within tumours, identified through FMISO PET CT, can enhance treatment outcomes in terms of LRC compared to standard treatment.

The secondary objectives include evaluating the impact of this treatment on overall survival, acute and late toxicity, and patient-reported outcomes such as quality of life. The study also aims to compare the outcomes between the dose-escalated group and the standard treatment group in a randomised controlled trial setting.

## Study/Trial design

DE-HyART is an open-label, parallel, randomised, single-centre, phase II study designed to evaluate the effectiveness and feasibility of dose escalation using FMISO PET scans for HNC. This superiority trial aims to assess the impact of integrating IMRT with simultaneous integrated boost (SIB) and dose escalation to hypoxic sub-volumes, as delineated by [18-F] FMISO, compared to standard-dose radiotherapy. The treatment protocol also incorporates concurrent chemotherapy with standard dosing of the physician’s choice and patients’ fitness. The protocol has been developed in accordance with SPIRIT guidelines [34, 35].

### Participants, interventions and outcomes

**Figure 1.**
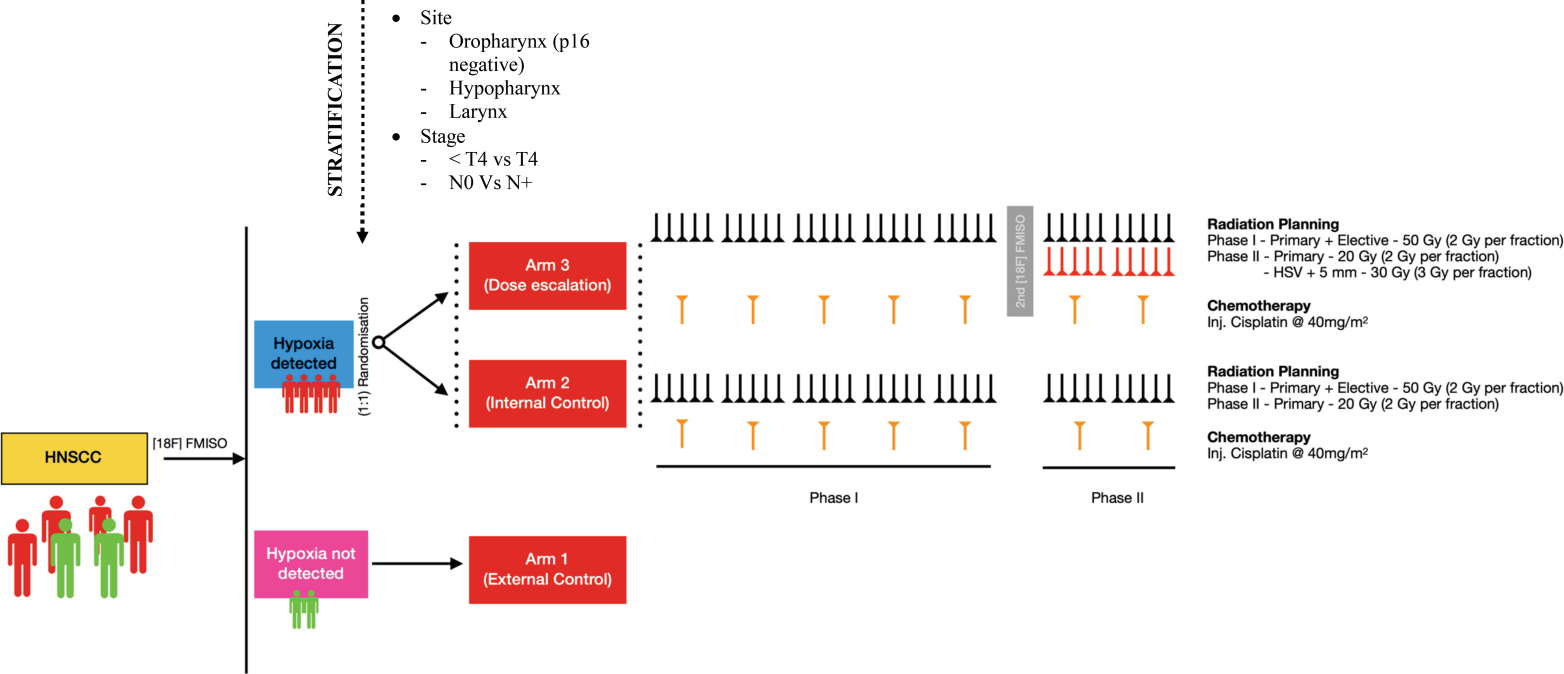
A detailed schematics showing flow of the study.

DE-HyART is an investigator-initiated study coordinated by the Department of Radiation Oncology at Rajiv Gandhi Cancer Institute and Research Centre, Delhi. The department will oversee the entire study, including registration, data management and radio-oncological quality assurance. The study has received approval from the Scientific Review Board and Institutional Review Board (IRB) for scientific and ethical compliance. All participants will provide written informed consent prior to enrollment, which will outline the randomised nature, scope and potential consequences of the study taken by the principal investigator or research coordinator. The flow of the study will be as mentioned in Table 1 and Figure 1.

### Eligibility criteria

All HNC patients planned for radical radiotherapy (± concurrent chemotherapy) in the department of radiation oncology were screened as per the inclusion and exclusion criteria mentioned in Table 2.

### Participant screening, recruitment and consent [18F] FMISO PET CT and hypoxia identification

All eligible patients will be subjected to screening [18F] FMISO scan, labelled as baseline FMISO. The primary challenge with using FMISO is its lipophilic nature, which slows its clearance from normal tissues such as blood and muscle, resulting in a low signal-to-background ratio. Numerous studies [12, 36–39] have evaluated FMISO parameters with clinical endpoints, demonstrating that a TMR ranging from 1.2 to 1.6 significantly correlates with the reduction of hypoxic volumes during the course of radiotherapy.

Therefore, a TMR of 1.4 will be employed to differentiate hypoxic from normoxic patients (TMR ≥ 1.4 and < 1.4 will be labelled as hypoxic and normoxic, respectively). Standardised assessment of SUVmax and SUVmean will be utilised. Tumour SUVmax will be determined by generating a region of interest (ROI) of 1 cm^2^ at the most metabolically active region of the tumour. For background reference, the contralateral sternocleidomastoid muscle at the caudal edge of the hyoid bone will be used, with a similar ROI to calculate the TMR.

### Timing of [18F] FMISO PET CT

All enrolled patients will undergo an [18F] FMISO PET CT scan. Patients exhibiting hypoxia, as determined by the PET results, will be randomised into either Arm 2 or Arm 3. A second [18F] FMISO PET CT scan will be performed between the fourth and fifth weeks of radiation therapy to evaluate the temporal variability of HSV.

## Arm allocation and randomisation

Depending upon the result of the baseline FMISO, the patient will be either hypoxic or normoxic. Patients exhibiting no hypoxia (TMR < 1.4) in their tumour will be labelled as Arm 1 and act as an external cohort. Patients with hypoxia (TMR ≥ 1.4) will be stratified by T stage, N stage and tumour site, then randomly assigned in a 1:1 ratio to either Arm 2 or Arm 3 using a computer-generated randomisation sequence to ensure balanced distribution across these variables. Both arms will be subjected to chemoradiation by IMRT and concurrent chemotherapy with cisplatin at 40 mg/m^2^. In Arm 3, the trial arm will receive an additional dose of 10 Gy @ 2 Gy per fraction in phase II (total 80 Gy) to the HSV + 5mm isotropic margin.

## Intervention

All patients will be treated using IMRT (with pre-specified image guidance protocol as per institutional standards).

## Arm 1

Patients will receive standard care and serve as external controls, representing typical clinical practice. Their cases will be reviewed in the head and neck multidisciplinary clinic, following the institutional approach. These patients will undergo treatment analogous to that of ‘Arm 2,’ but with allowances for protocol deviations at the discretion of the treating radiation oncologist.

## Arm 2

The prescribed radiotherapy dose will be 70 Gy, delivered in daily fractions of 2 Gy. The elective volume will receive 50 Gy, administered in 2 Gy daily fractions over the first 5 weeks. This will be followed by a boost to the high-risk volume, delivering an additional 20 Gy in 2 Gy daily fractions over the subsequent 2 weeks. The entire treatment will be delivered in a phased approach using sequential planning.

## Arm 3

**Figure 2.**
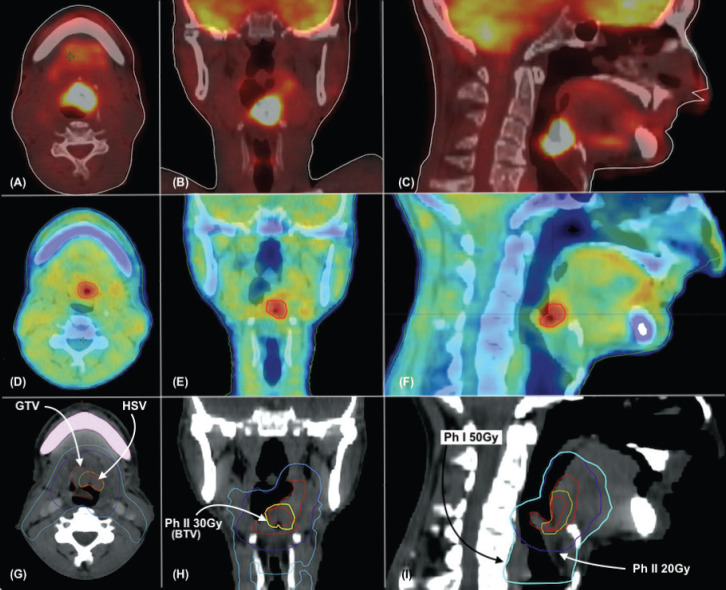
(a–c) shows 18F FDG PET CT images in axial, coronal and sagittal views, respectively. (d–f) shows 18F FMISO PET CT images in axial, coronal and sagittal views, respectively. (g–i), target delineation as labeled in axial, coronal and sagittal views, respectively. GTV – Gross Tumour Volume, HSV – Hypoxic Sub-Volume, Ph I 50 Gy – Volume receiving 50 Gy in 25 fractions in phase 1, Ph II 20Gy – Volume receiving 20 Gy in 10 fractions in phase 2. Ph II 30Gy – Volume receiving 30 Gy in 10 fractions in phase 2.

The radiation dose will be similar to ‘Arm 2’. In addition, the HSV identified on baseline FMISO scans will be contoured and an isotropic margin of 3 mm will be given. This volume will be boosted in phase II to a total dose of 80 Gy. (Addition of 30 Gy in 3 Gy daily fraction added in phase II as a simultaneous integrated boost – SIB) (Figure 2). The BED and EQD2 for Arm 3 (to HSV) are 99 Gy_10_ and 82.5Gy, respectively, in comparison to 84 Gy_10_ and 70 Gy, respectively, in Arm 2.

## Contouring protocol

The patients will be immobilised using a four-clip thermoplastic cast (Orfit Ray™ Cast) as per standard institutional protocol. The treatment planning scans will be performed with IV contrast and 3 mm slice thickness. Daily IGRT is not mandatory but recommended. The daily shift should be at most 5mm of the planning target volume in any axes.

Arm 2 and 3: Gross tumour volume (GTV) will be delineated using MRI and FDG PET CT per standard institutional standards. Clinical target volume (CTV 1) will include GTV based on clinical and radiologic information (including primary tumour and involved lymph nodes), expanded with isotropic 5-mm margins. Clinical target volume (CTV 2) will consist of the high-risk areas harbouring microscopic disease and elective nodal regions. CTV 2 will be created individually for the primary target and the secondary lymphatic. A 5 mm margin will be added to each CTV 1 and 2 to form planning target volume (PTV) 1 and 2, respectively. A model dose of 50 Gy in 25 fractions will be prescribed to PTV 2 in phase I and 20 Gy in 10 fractions to PTV 1 in phase II.

Hypoxic sub-volume delineation (Arm 3): The HSV delineation will be done for patients in arm 3 using baseline FMISO. The HSV will be contoured as per baseline FMISO and adjusted according to the second FMISO scan done between the fourth and the fifth week of radiation treatment. A planning CT will also be repeated at the time for adjusting the HSV to account for temporal changes. The HSV, thus, delineated will be isotropically enlarged by 3 mm to form a biological target volume (BTV). The BTV will be truncated from bone, air and organs at risk (OAR) by 3mm. The BTV will be prescribed 30 Gy in 10 fractions.

Adjusting HSV in phase 2*:* The HSV will not be adapted based on the response, but will be adjusted only to account for geometrical changes due to image registration in the new scan taking into account – OARs, air and bone. The boost volume will primarily remain consistent with the baseline HSV.

Organ at risk: Dose constraints to OAR and planning risk volume will be taken from quantitative analysis of normal tissue effects in the clinic (QUANTEC) and RTOG protocols (wherever QUANTEC did not provide constraints). The patients in ‘Arm 3’ will be subjected to isotoxic dose escalation, with constraints remaining the same as Arm 2.

Treatment planning: All patients will be treated on TrueBeam™ version 1.6 (Varian Medical Systems Inc., Palo Alto, CA, USA) equipped with HD-120 leaf MLC (28 pairs of 5 mm and 32 pairs of 2.5 mm). Plans were made on Varian Eclipse™ Version 15 (Varian Medical Systems Inc., Palo Alto, CA, USA) using 7–9 co-planar static fields with collimator angle 0. IMRT plans will be optimised using a dose-volume optimiser and calculated using the Anisotropic Analytical Algorithm.

## Adherence to protocol and concomitant care

Patients will be monitored weekly to assess their tolerance to treatment. Any patient exhibiting grade 3 or higher toxicity will require urgent therapeutic intervention. The most anticipated side effects are mucositis and dysphagia. Patients will be evaluated weekly for the need for nutritional support and feeding tube placement, as well as on an as-needed basis. In rare instances of severe toxicities not responding to conservative measures, a treatment break may be necessary. The resumption of radiotherapy will be contingent upon the resolution of major toxicities, with the aim of restarting radiotherapy as soon as clinically feasible.

Outcome measures: Oncological outcomes will be collected by a designated team of head and neck specialists (radiation oncologist, surgeon and radiologist)

Response assessment*:* The patients would be assessed for the first time after 12 weeks of completion of definitive radiotherapy. The patients would be assessed by 18-FDG PET CT (± MRI) and direct laryngoscopy. The need for biopsy versus repeat imaging at a short interval will be taken by a multispeciality clinic taken into view the clinical examination, laryngoscopy findings (including narrow band imaging) and radiological findings.

*Primary endpoint: locoregional control* (LRC): LRC is defined as the absence of tumour recurrence or progression within the primary tumour site and the regional lymph nodes, as determined by clinical evaluation, imaging studies and/or biopsy confirmation. LRC will be assessed at predefined time points, with the primary time point being 2 years post-treatment. Locoregional recurrence within 2 years post-treatment is associated with significant morbidity and reduced quality of life. Therefore, 2-year LRC serves as a clinically meaningful endpoint to guide treatment decisions and optimise outcomes for patients receiving escalated radiation doses.

### Secondary endpoints

*Overall Survival (OS)*: The duration of OS is defined as the time from the date of randomisation to death from any cause. If there is no death, the OS duration will be censored at the time of analysis.

*Locoregional relapse-free survival (LRFS)*: LRFS is defined as the time from the date of randomisation to the first histopathologically confirmed relapse of locoregional disease. If there is no confirmed recurrence, the LRC duration will be censored at the time of analysis. Death from any cause will be considered as an event in LRFS.

*Acute toxicity:* Acute adverse events will be assessed and recorded according to the NCI CTCAE version 5.0 [40]. Evaluations will be conducted at baseline, mid-treatment (week 4), immediately post-treatment, 2 weeks post-radiotherapy and subsequently at 3 months post-treatment.

*Late toxicity assessment*: Long-term toxicities of the upper aerodigestive tract will be recorded at 1 and 2-year follow-ups. They will be assessed using the RTOG long-term toxicity assessment [41].

*Patient-reported outcome*: The data will be collected by the trial coordinator and, research nurse by collecting proformas filled by patients.

European Organisation for Research and Treatment of Cancer (EORTC) head and neck questionnaires: The Hindi versions of the EORTC QLQ-C30 [42] and QLQ-H and N35 questionnaires [43, 44] will be obtained from the QoL unit, EORTC Data Centre in Brussels, Belgium. The EORTC QLQ-C30 questionnaire consists of five functional scales that include physical, role, cognitive, emotional and social; three symptom scales that include fatigue, pain and nausea/vomiting; a global QoL scale; and six single items that include dyspnea, insomnia, appetite loss, constipation, diarrhoea and financial difficulties. The QLQ-H and N 35 questionnaires consisted of seven multiple-item scales that assess the symptoms of pain, swallowing ability, senses (taste/smell), speech, social eating, social contact and sexuality and six single-item scales that assess the presence of symptomatic problems associated with teeth, mouth opening, dry mouth (xerostomia), sticky saliva, coughing and feeling ill. All the scales of the EORTC QLQ-C30, QLQ-H and N35 range from 0 to 100.

Dysphagia assessment: Dysphagia is commonly evaluated through functional assessments of swallowing, such as videofluoroscopy or fiberoptic endoscopic evaluation. Furthermore, tools that quantify the severity and impact of dysphagia are essential for determining treatment outcomes and rehabilitation requirements. Various instruments are available for assessing dysphagia, including the 10-item Eating Assessment Tool [45], the Sydney Swallow Questionnaire [46], the Swallowing Quality of Life Questionnaire (SWAL-QOL) [47] and the MD Anderson Dysphagia Inventory (MDADI) [48]. The MDADI includes global, emotional, functional and physical subscales, each designed to capture different aspects of how dysphagia impacts quality of life. The global assessment question provides a straightforward method for evaluating overall dysphagia severity. It was specifically chosen because of its relatively shorter 20-point questionnaire and easier implementation, especially when compared to SWAL-QOL or dysphagia handicap index [49].

## Sample size

Estimated LC rates 59% and 84% in the control and treatment arm, respectively, in the phase II study by Welz *et al* [29]. For achieving an 80% power (i.e., 1−β = 0.8) at the 5% level of significance (i.e., α = 0.05) with the equal allocation (i.e., k = 1), the sample size for Arm 2 and 3 is 62 patients in each arm. This study is designed as a Phase II trial aimed at evaluating the preliminary efficacy and safety of the intervention. As such, the primary focus is on detecting a signal of LRC rather than establishing a long-term survival benefit. The sample size of 124 participants (62 per group) is consistent with Phase II study designs, which typically involve smaller cohorts to efficiently assess early efficacy. Due to the exploratory nature of this trial, the follow-up period was set at 2 years to capture early control rates, which are increasingly recognised as predictive of long-term outcomes in salivary ductal cancers. The 2-year endpoint aligns with the study’s goal of generating data to inform the feasibility of subsequent Phase III trials.

### Data collection and analysis

All data will be collected in the central registry of the institute and anonymised. All statistical analyses were performed using the Statistical Package for Social Science System (SPSS v26, SPSS Inc, Chicago, IL) or equivalent software. Continuous variables will be written as mean (along with standard deviation) and median (along with interquartile range). Categorical variables will be represented as frequencies and percentages. Inter-cohort testing will be performed using chi-square for categorical variables and independent-t/nonparametric tests for continuous variables. Time-to-event endpoints will be measured from the date of randomisation to the date of failure or last follow-up. The LRC rate at 2 years will be estimated using the Kaplan–Meier method, with the time to locoregional failure defined as the duration from the start of radiation therapy to the first documented recurrence or progression within the primary tumour site or regional lymph nodes. The LRFS and OS, as mentioned earlier, death from any cause will be considered a failure. The LRFS and the OS rates will be estimated using the Kaplan–Meier product-limit method for each arm. Their distributions will be compared between treatment arms (Arms 2 and 3) with a two-sided log-rank test ≤ 0.05. The clinicopathological characteristics, survival outcomes and treatment modalities will be analysed using the univariate and multivariate Cox proportional hazards model. All eligible patients will be included in the analysis in the arm to which they were randomly assigned (intent-to-treat).

### Interim analyses

Interim analyses are not planned for this study to avoid the potential introduction of Type I and Type II errors. Conducting interim analyses could increase the likelihood of false-positive results, leading to incorrect conclusions about the treatment’s efficacy. Additionally, it could result in false negative findings, potentially overlooking beneficial effects that emerge later in the treatment course.

### Oversight and data monitoring committee

Organisational structure, protocol revision, publication and managing general administrative function will be carried out by principal investigator and co-principal investigator. A separate trial committee (see title page for members) will be tasked to ensure quality checks on the radiation plan, data entry and 6-monthly report and audit. Any unexpected or grade 5 toxicity will be reported to the Serious Adverse Events (SAEs) Committee, which will monitor and evaluate the potential causes of these adverse effects.

Recruitment strategy: The trial will be conducted at a tertiary care centre specialising exclusively in oncology. Based on the annual case volume managed by the head and neck multispecialty clinic, the projected sample collection is expected to be completed within 2 years.

Retention strategy: Patients will be advised on the importance of maintaining diligent follow-up and completing the health-related quality of life questionnaire. In the event of a missed follow-up, efforts will be made to contact patients via telephone, including offering video consultations, to ensure the completion of the required forms. Long-term care will be provided to those whose side effects are considered to be beyond standard of care treatment, as assessed by the SAE committee.

### Trial status

The trial is currently in the recruitment phase, actively enrolling patients since March 2024, with an expected completion date of March 2026. The protocol in use is version 1.2, dated August 28, 2023.

## Discussion

The trial is currently ongoing and in the recruitment phase, with completion of recruitment anticipated by March 2026. A significant challenge for the trial is the procurement of the FMISO radiotracer, which may potentially delay the project timeline. To date, most patients are tolerating the treatment well, with no serious SAEs reported.

## Ethics approval and consent to participate

The study is being conducted following approval from the IRB and the Scientific Review Board. Before enrollment, informed consent forms, signed by the patients, will be collected by the principal investigator, research coordinator or an assigned researcher. All data will be anonymised, with no patient identifiers retained in the research database. Additionally, a separate clinical data record will be maintained in the institute’s registry. Any amendments to the protocol will be promptly reported to the IRB and the funding agency.

## Conflicts of interest

The authors have no competing interests.

## Funding

The study has been funded by Varian Medical Systems, Inc.

## Consent for publication

Yes, including for ancillary studies if planned.

## Availability of data and material

Individual patient data and statistical analysis required to support the protocol can be provided upon request and the final decision to share will be subject to steering committee approval. The investigator and the participating site will ensure data protection and prevent any disclosure outside the scope of the research.

## Author contributions

ST and MG (Munish Gairola) have contributed equally to all aspects of the protocol, study design, writing, conceptualisation and are the co-PI and PI of the study, respectively. ST, MV, PA and AN have contributed to constructing the statistical design for the study. MG (Munish Gairola), MM, PSC and MG (Manoj Gupta) have been involved in analysing PET metrics and segmentation. SR, AB (Akash Bellige), AS, VY, VA, DKS, IA and RS have helped in reviewing the literature and designing proper design parameters. KSC, JSS, AP, MA, AA, RB and AKD have maintained the quality check on trial study parameters. SS, DV, AB (Abhishek Bhadri) and HV have maintained radiation quality check and control. All authors have read and contributed to various aspects of patient care, protocol implementation and manuscript writing.

## Figures and Tables

**Table 1.** Schedule of enrolment, interventions, and assessments for the DEHyART study as per SPIRIT guidelines.

Time points	Study period														
	Enrolment	Allocation	Post allocation (During RT)								Acute phase (Post RT)		Late phase (Post RT)		
	Pre RT	Pre RT	Week								Month		Month		
			1	2	3	4	5	6	7	Post RT 2	3	6	12	18	24
Enrolment															
Eligibility screen	X														
Informed consent	X														
Diagnostic/staging workup	X														
CCI	X														
F MISO PET CT (baseline)	X														
Allocation															
Randomisation		X													
Intervention															
Arm 1 (CRT)			X	X	X	X	X	X	X						
Arm 2 (CRT)			X	X	X	X	X	X	X						
Arm 3 (CRT)			X	X	X	X	X	X	X						
Dose escalation (Arm 3)							X	X	X						
Second F MISO PET CT (Arm 3)						X									
Assessment															
CTCAE v5 (acute toxicity)		X				X			X	X	X	X			
EORTC QLQ C30 and HN35		X				X			X	X	X	X	X	X	X
MDADI		X				X			X	X	X	X	X	X	X
RTOG (late toxicity)													X	X	X
Response assessment (Clinical ± radiological)											X	X	X	X	X

**Table 2:** Inclusion and exclusion criteria for enrolment in DEHyART study.

Inclusion criteria	Exclusion criteria
Age: 18–70 years Willingness to sign informed consent (written/video documentation) Performance status: ECOG 0 – 2 Histology proved – squamous cell carcinoma Any grade, gender Tumour sites: Oropharynx, Hypopharynx and Larynx Sufficient bone marrow reserve within the last 14 days. Hb: > 10 g/dl (corrected) TLC: > 4,000 per cumm Platelet: >1.5 Lakhs per cumm Liver functions and kidney functions within normal limits Nutritional and dental assessment before inclusion into the study	HPV (p16) positive tumours Prior surgery and/or radiation therapy given for any HNC T1/T2 Glottis Metastatic disease or disease not amenable for definitive locoregional treatment. Medical co-morbidity hampering the administration of radiation and/or chemotherapy Pregnancy or lactation
